# Effect of PUVA and NB-UVB Therapy on the Skin Cytokine Profile in Patients with Mycosis Fungoides

**DOI:** 10.1155/2022/3149293

**Published:** 2022-02-21

**Authors:** Arfenya E. Karamova, Dmitry A. Verbenko, Anastasiia A. Vorontsova, Maryana B. Zhilova, Alexandr A. Nikonorov, Eugenia R. Gatiatulina, Ludmila F. Znamenskaya, Alexey A. Kubanov

**Affiliations:** ^1^State Research Center of Dermatovenereology and Cosmetology, Korolenko St., 3, Bldg 6, 107076 Moscow, Russia; ^2^RSDC (Russian Society of Dermatovenereologists and Cosmetologists), Moscow, Russia; ^3^All-Russian Research Institute of Medicinal and Aromatic Plants (VILAR), Grina St., 7, 117216 Moscow, Russia

## Abstract

**Background:**

Mycosis fungoides (MF) is the most common subtype of cutaneous T-cell lymphoma. The aim of the present study was to produce up-to-date information on different phototherapy approaches on skin cytokines in patients with MF.

**Methods:**

A total of 27 patients with mycosis fungoides were treated with phototherapy: NB-UVB (narrow‐band ultraviolet B therapy) (10 patients) and PUVA (long-wavelength ultraviolet radiation of spectrum A with the use of skin-photosensitizing furocoumarins) therapy (17 patients). Evaluation of the effectiveness of treatment was carried out using BSA (body surface area) and the modified assessment of the severity of the skin lesions scale (mSWAT) used to quantify tumor mass in cutaneous T-cell lymphomas. Average numbers of procedures were 30.2 and 27.8 in the NB-UVB and PUVA groups, respectively. The median total dose of NB-UVB irradiation was 19.9 J/cm^2^ and PUVA therapy was 104.0 J/cm^2^. The overall response to therapy including complete and partial remission was 74.9% in the total group; 70% in the NB-UVB group, and 77.7% in the PUVA therapy group. In the obtained biopsies from lesions, surrounding tissue before treatment and skin samples of four healthy volunteers, the concentration of the IL-1*β*, IL-4, IL-6, IL-10, IL-17A, IL-17F, IL-21, IL-22, IL-23, IL-25, IL-31, IL-33, IFN-*γ*, sCD40L, and TNF-*α* cytokines was studied. An increase in IL-4 and TNF-*α* levels was shown in the lesional skin of patients compared to the skin of healthy controls. After the treatment, positive correlations of mSWAT with the levels of IL22, IL33, and TNF-*α* in the tumor tissue were found. The levels of IL10 and IFN-*γ* after PUVA treatment were increased in comparison to baseline. There was no difference in cytokine levels before/after NB-UVB therapy.

## 1. Introduction

Mycosis fungoides (MF) is the most frequent subtype of skin T-cell lymphoma [1] with monoclonal proliferation of neoplastic CD4+ T cells in the skin [2]. Cytokines were intensively tested by various methods in all types of lymphoma including MF. The Th1 cytokine profile prevails in the early stages of MF, while at the late stage, the immune environment changes the Th1-type cytokine pattern to Th2-type. Excessive production of Th2 cytokines contributes to itching, reduces Th1 responses, and increases the susceptibility to infection, the leading cause of death in patients with the late stages of MF [3]. IL4 and IL13 act as suppressors of\do suppress the expression of Th1-type cytokines and stimulate the proliferation of malignant cells [4]. In contrast to Th1 and Th2 cytokine patterns, the Th17 phenotype was observed at all stages of MF. At the same time, the role of the Th17 profile and IL17 in MF is controversial [5]. Some studies have shown high expression of IL17 associated with MF progression, [6, 7] while others reported low levels of IL17 [4, 8]. Krejsgaard et al. demonstrated that proinflammatory IL17 activates the JAK3/STAT3 signaling pathway that contributes to the progression of cutaneous T-cell lymphoma [7]. In that way, the cytokine environment substantially determines the prognosis of the disease by affecting the phenotype and functional properties of skin T-lymphocytes [9].

The early stages of cutaneous T-cell lymphoma (MF) can be managed with skin-targeted therapy [10, 11]. Recent studies have demonstrated the high efficacy of NB-UVB and PUVA in MF [12]. At the same time, the data on cytokine profile at various stages and severity of MF when exposed to phototherapy are insufficient and contradictory.

In this regard, the study aimed to assess the influence of different phototherapy approaches on the cytokine proteome profiling of the skin in patients with MF and to determine the reliable cytokines for predicting disease progression with emphasis on the potentially most important.

## 2. Materials and Methods

An open, uncontrolled prospective study was conducted in accordance with the Declaration of Helsinki of 1975 and its later amendments. A total of 27 patients with mycosis fungoides in the early stages (IA-IIA) and 4 healthy volunteers participated in the present study. The protocol of the study was approved by the local Ethics Committee (protocol No. 2 28.02.2018). All participants provided written informed consent.

### 2.1. Treatment Regimen

Treatment was performed by narrow‐band ultraviolet B therapy (NB-UVB) (*n* = 10) or long-wavelength ultraviolet radiation of spectrum A (PUVA) (*n* = 17) using Waldmann UV7002К cabin (Herbert Waldmann GmbH & Co. KG, Villingen-Schwenningen, Germany). Fruits of *Ammi majus L.* as a furocoumarin source were prescribed in 20 mg tablets at a dose of 0.8 mg/kg of body weight given 2 hours before irradiation.

The course of treatment was 20–40 procedures (depending on the individual response to therapy, four times per week) with the initial doses of UVA 0.25–1.0 J/cm^2^ (based on the skin phototype) increased every second procedure by 10–30% or 0.25–1.0 J/cm^2^; the initial dose of NB-UVB 0.1–0.2 J/cm^2^ (based on the skin phototype) was increased every second procedure by 0.1 J/cm^2^.

Evaluation of the treatment effectiveness was carried out using the BSA index (body surface area of the involved skin), mSWAT scale to quantify tumor mass in cutaneous T-cell lymphoma, and the criteria proposed by the International Society for Cutaneous Lymphomas (ISCL). The European Organization for Research and Treatment of Cancer (EORTC), and the American Cutaneous Lymphoma Consortium (USCLC) [13].

BSA index was calculated using the following formula [1]:

(1) $$\begin{array}{c} \text{BSA}\left(\%\right)=0.1\text{ Sh}+0.2\text{ Su}+0.3\text{ St}+0.4\text{ Sl,} \end{array}$$

where S is the body area involved; h, head; u, upper limbs; t, trunk; and l, lower limbs.

### 2.2. Sample Preparation

Tissue samples with the 5 × 5 mm size were obtained from the tumor and surrounding skin of patients and healthy volunteers. Biopsies were weighed with milligram precision, transferred to special disposable Medicons chambers (Becton Dickinson, USA), and then fragmented. Homogenization was performed using the Medimachine automated tissue disaggregation system (Becton Dickinson, USA) for 2 min with the addition of 1 ml of phosphate buffer (pH = 7.4) followed by filtration through a 50 *μ*m filter. The end-product suspensions were frozen and stored at –80°С until further multiplex immunological analysis was performed.

### 2.3. Determination of Cytokine Concentration

Assessment of the cytokine concentrations (IL-1*β*, IL-4, IL-6, IL-10, IL-17A, IL-17F, IL-21, IL-22, IL-23, IL-25 (IL-17E), IL-31, IL-33, IFN-ү, sCD40L, and TNF-*α*) was performed on a BioPlex200 (Bio-Rad, USA) using Bio-Plex Pro Human Th17 Cytokine Panel 15-Plex (Bio-Rad) kit as described earlier [14]. The study was performed in duplicates using an automatic microplate washing station Bio-Plex Pro Wash Station (Bio-Rad) and a microplate shaker (Biosan). A calibration kit (Bio-Rad) was used for calibration. The results were automatically recorded by Bio-Plex Manager software (Bio-Rad) and expressed in pg/mg of tissue.

### 2.4. Statistical Analysis

The analysis and visualization of the data obtained were carried out using RStudio for MacOS (version 1.3.1056). Data normality was estimated by the Shapiro–Wilk test. Data were presented as median (25–75). To assess the differences between groups, the Kruskal–Wallis test was used, followed by Dunn's post hoc test for multiple comparisons. Due to the non-Gaussian distribution of data, the Wilcoxon test for paired comparisons was used to assess the differences in cytokine levels before and after the treatment. Spearman's correlation analysis and correlogram were used to evaluate the correlation between cytokine levels. Differences were considered significant at *p* < 0.05.

## 3. Results

### 3.1. Skin Cytokine Levels before the Treatment

First, we analyzed data on the level of cytokines in samples of tumor tissue and surrounding tissue in patients with MF before treatment and compared it with healthy controls. Significant increase in IL4 (control/tumor tissue) (*p*=0.025) and TNF-*α* (control/tumor tissue) (*p*=0.012) levels was revealed (Table 1). Original data and the characteristics of the patients are presented in Supplementary Table 1S.

### 3.2. Phototherapy Effect on the Severity of the Disease

One out of 27 patients developed disease progression, one patient did not respond to treatment, and one patient experienced adverse events during therapy.

**Figure 1:**
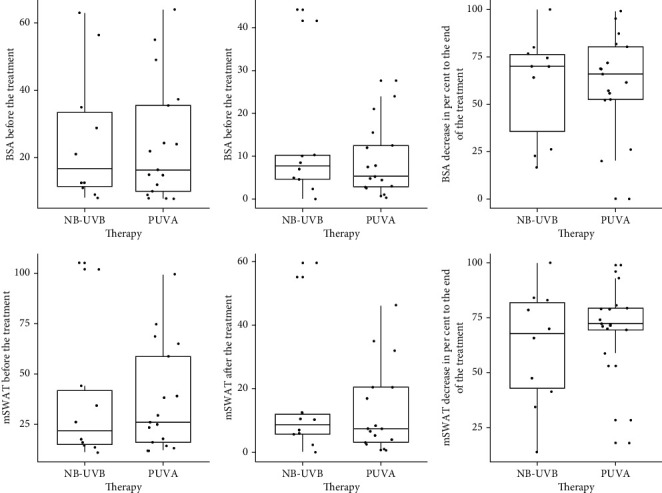
Effectiveness of different treatment approaches in patients with mycosis fungoides.

After treatment, the mSWAT score was 7.45 (3.1–20.5) in patients in the PUVA therapy group and 8.65 (5.6–12.5) in the NB-UVB therapy group (Figure 1). The percentage of mSWAT decrease in mSWAT in the PUVA group was 4.5% higher than in the NB-UVB group and amounted to 72.3 (69.4–79.3) vs 67.8 (41.3–82.9), respectively. However, none of these differences were significant. The total dose was 104.0 (35.0–160.0) J/cm^2^ in the PUVA therapy group and 19.92 (14.4–22.8) J/cm^2^ in the NB-UVB therapy group.

After the treatment, six patients from the NB-UVB therapy group achieved partial remission, one, complete remission, and two, stabilization. In one case, no treatment effects were observed. All patients of the PUVA therapy group have demonstrated the effectiveness of treatment: one patient achieved complete remission, 13, partial remission, two, stabilization, and one patient, disease progression. Original data and the characteristics of the patients are presented in Supplementary Table 2S.

### 3.3. Skin Cytokines after Treatment

Interestingly, the PUVA therapy was accompanied by an increase in the level of IL10 and IFN-*γ* by 10% (*p*=0.021) and 4.75 times (*p*=0.038) in the tumor tissue, respectively (Table 2). However, there were no similar dynamics in the NB-UVB group. Considering the significant individual variations in the cytokines levels of tumor tissue and the orphan nature of the disease, Supplementary Table 3S presents the cytokine profile of patients with MF.

Analysis of cytokine levels revealed two patients with pronounced differences from the main sample (Supplementary Tables 2S and 3S). These patients were treated with NB-UVB (total dose of 2.1 and 22.8 J/cm^2^). Patient L9 had very high levels of IL33 and IL22 (almost ten times higher), and TNF-*α* was 7.2, and IL4 was nearly five times higher than the average. In the clinical picture, thin plaques covering 56.4% of the skin were dominated. The mSWAT score after treatment was also above average: 59.70 vs 13.61 (stabilization achieved). Patient L10 had very high concentrations of IL31, sCD40L, IL17F, IL23, IFN-*γ*, IL10, and IL4 and significantly differed in cytokine profile from the main sample. Lesions were localized on the skin of the buttocks and groin area with the transition to the skin of the thighs. They were mainly represented by spotty foci with signs of superficial atrophy. The mSWAT score after treatment was 10.5; stabilization was achieved.

**Figure 2:**
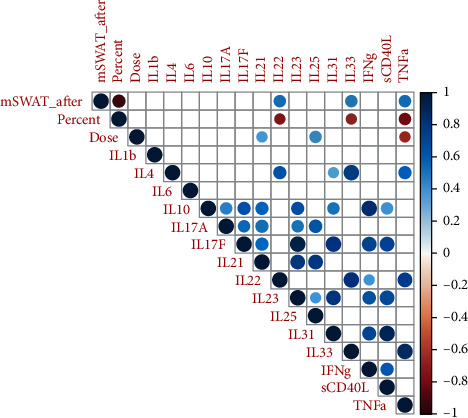
Correlation matrix of the relationship between the cytokine levels in tumor tissue in patients with MF after treatment. Only significant correlations are shown (*p* < 0.05). Blue indicates a positive relationship and red a negative relationship. The intensity of the color and the size of the circle are proportional to the correlation coefficients. The legend color shows the values of the correlation coefficients.

Correlation analysis before treatment did not reveal any relationship between mSWAT and skin cytokines (data not shown). However, mSWAT after treatment was positively associated with the level of IL22 (*r* = 0.59, *p*=0.006), IL33 (*r* = 0.56, *p*=0.010), and TNF-*α* (*r* = 0.59, *p*=0.007) (Figure 2).

## 4. Discussion

Type 2 (Th2) T-helper cytokines, especially IL4, IL5, IL10, and IL13, are significant in MF development [15, 16]. In our study, increased levels of IL4 and TNF-*α* were found in lesions of patients with MF compared to healthy controls (Supplementary Table S1). These findings are in line with the data indicating the high IL4 production by Th2 lymphocytes in T-cell lymphomas. At the same time, there is evidence that it is uncharacteristic for the initial stages of the disease (IA-IIA) considered in our study and an increase in IL4 produced by Th2 cells more often occurs at the later stages of MF [17]. Th1/Th2 dysfunction plays a key role in lymphomagenesis and the shift from Th1 to Th2 phenotype is accompanied by disease progression [18].

The role of TNF-*α* in the pathophysiology of MF is believed to be complex [19]. TNF-*α* can induce epidermotropism via interferon-inducible protein (IP-10) [20] and acts as an autocrine growth factor [21]. It is a central mediator of immune and inflammatory responses, and the study by Amitay-Laish et al. showed the possibility of MF exacerbation by anti-TNF-*α* therapy [22]. In an *in vitro* study of cutaneous T-cell lymphoma, Wu and Wood revealed the induction of extrinsic apoptosis by gentian violet via the Fas and TRAIL (TNF-related apoptosis-inducing ligand) pathways [23].

A number of studies have shown that the tumor tissue in MF contains an increased amount of IL17 and IL22, and the rise in IL17 expression is associated with the disease progression [6, 7]. At the same time, a recent study by Papathemeli et al. revealed a contradictory pattern with the downregulation of IL-17A and IL-17F and upregulation of IL-22 in the skin of MF patients [24]. The findings of the current study do not support the mentioned data: we failed to find significant changes in IL17 and IL22 levels in tumor tissue vs surrounding skin tissue of patients. However, after the UV therapy, a negative correlation of IL22 with the percent of mSWAT decrease was established (Figure 2).

After the treatment, patients undergoing PUVA therapy had increased IL10 and IFN-*γ* levels, which was not observed in the NB-UVB group. The revealed increase in IFN-*γ* after PUVA therapy is not in line with the existing data. According to Grewe et al., successful therapy (UVA-1 or UVA/UVB) of atopic dermatitis was associated with a decrease in IFN-*γ* expression level [25]. In a study by Rácz et al., the use of NB-UVB in the treatment of psoriasis was accompanied by the downregulation of IFN-*γ* and Th17 pathways [26]. Furthermore, there is evidence that IFN-*γ* production by Th1 is reduced under the influence of IL10 [27, 28]. However, an increase in IFN-*γ* is undoubtedly a positive aspect of PUVA therapy, as it can be considered a reverse of the MF-induced shift from Th1 to a Th2 cytokine profile. It may indicate stabilization or even some regression of the pathological process. This suggestion is confirmed by the high efficiency of IFN-*γ* in patients with IA–IIIA stages of MF [29] and in pilot phase II clinical trials [30].

The increase in IL10 during PUVA treatment is in line with the existing data on the induction of keratinocyte-derived IL10 expression by UV [31, 32]. The lack of a similar effect in the NB-UVB group may be related to the mechanism of action: as already mentioned, UVB mainly affects Langerhans cells and epidermal keratinocytes, while UVA penetrates deeper into the dermis and affects dendritic cells, dermal fibroblasts, endotheliocytes, and inflammatory cells, T cells, mast cells, and granulocytes [24, 32]. It is also possible that the toxic products produced by PUVA are longer-lived than those of UVB, resulting in a sustained downstream immunosuppressive cascade [33].

Noteworthy is the positive correlation of mSWAT after phototherapy with the level of IL22, IL33, and TNF-*α*. It is possible to consider them as a prognostically unfavorable and an important issue for future research.

In our study, the overall response to therapy (partial and complete remission) in the total group was 74.9%, in the NB-UVB group, 70%, and in the PUVA group, 77.7%. The achievement of complete remission was 5.5% in the PUVA group and 10% in the NB-UVB group and partial remission was observed in 72.2 and 60% of patients, respectively. In general, our data are consistent with the results of a retrospective study on 778 patients with MF at stage IA-IIA, indicating an absence of difference between the overall response in the PUVA and NB-UVB groups [34].

Summarizing the obtained results, the following have been demonstrated:

1. The use of PUVA and NB-UVB is effective for the treatment of the early stages of MF.
2. Differences in IL4 and TNF-*α* cytokine levels were revealed when comparing tumor tissue in patients with MF with healthy volunteer samples. Together with that, studied cytokine levels showed no difference in the levels at tumor tissue vs surrounding tissue of MF patients.
3. An increase in IL10 and IFN-*γ* concentrations was revealed in patients undergoing PUVA therapy. However, there were no differences in cytokine levels before/after treatment in patients with NB-UVB.

## Figures and Tables

**Table 1:** Cytokine levels in patients with mycosis fungoides compared to healthy controls.

Cytokines	Healthy controls (*n* = 4)	Patients with mycosis fungoides (*n* = 21)	
		Tumor tissue	Surrounding tissue
IL1b	0.04 (0.0225–0.0725)	0.48 (0.158–0.766)	0.18 (0.1–0.94)
IL4	0.04 (0–0.09)	0.28 (0.11–0.65)	0.49 (0.14–0.94)^*∗*^
IL6	0.00 (0–0.005	0.02 (0–0.03)	0.02 (0–0.22)
IL10	0.01 (0–0.04)	0.00 (0–0.125)	0.00 (0–0.091)
IL17A	0.07 (0.015–0.142)	0.09 (0–0.17)	0.06 (0.01–0.15)
IL17F	0.17 (0.128–0.24)	0.00 (0–0.432)	0.00 (0–0.231)
IL21	0.00 (0–0.115)	0.45 (0–1.67)	0.19 (0.12–1.02)
IL22	0.31 (0.165–0.43)	0.07 (0.0005–0.705)	0.65 (0.04–1.8)
IL23	0.08 (0.06–0.22)	0.00 (0–0.572)	0.00 (0–0.045)
IL25	0.02 (0–0.04)	0.01 (0–0.065)	0.01 (0–0.05)
IL31	0.85 (0–2.08)	0.09 (0–1.51)	0.43 (0.04–1.7)
IL33	16.54 (11.10–26.40)	17.52 (7.91–116.0)	60.32 (10.1–133.0)
IFN-*γ*	0.06 (0.0225–0.132)	0.05 (0–0.205)	0.03 (0–0.06)
sCD40L	0.16 (0.045–0.363)	0.27 (0.110–0.552)	0.30 (0.13–1.79)
TNF-*α*	0.01 (0–0.04)	0.18 (0.011–0.3)	0.23 (0.2–2.55)^*∗*^

Data are presented as median (25–75). ^*∗*^Significant difference in comparison with healthy controls (*p* < 0.05).

**Table 2:** Cytokine concentrations in tumor tissue before and after treatment compared to healthy controls.

Cytokines	Healthy control (*n* = 4)	PUVA		UVB311	
		Before (*n* = 15)	After (*n* = 15)	Before (*n* = 6)	After (*n* = 5)
IL1b	0.045 (0.023–0.073)	0.39 (0.075–0.96)	0.19 (0.07–0.405)	0.14 (0.1–0.442)	0.29 (0.2–2.76)
IL4	0.04 (0–0.09)	0.46 (0.095–1.28)	0.36 (0.175–0.919)	0.68 (0.526–0.805)^†^	1.27 (0.49–4.3)
IL6	0.00 (0–0.005	0.02 (0.01–0.379)	0.05 (0.025–0.11)	0.00 (0–0.047)	0.01 (0–0.08)
IL10	0.01 (0–0.04)	0.00 (0–0.093)	0.10 (0–0.382)^*∗*^	0.00	0.00 (0–0.43)
IL17A	0.07 (0.015–0.142)	0.09 (0.025–0.175)	0.11 (0.031–0.326)	0.00 (0–0.047)	0.02 (0–0.24)
IL17F	0.17 (0.128–0.24)	0.00 (0–0.206)	0.40 (0–0.830)	0.00 (0–0.758)	0.00 (0–1.37)
IL21	0.00 (−0.115)	0.35 (0.138–1.06)	2.31 (0.156–6.40)	0.10 (0–0.242)	0.20 (0–17.1)
IL22	0.31 (0.165–0.43)	0.65 (0.075–1.66)	0.57 (0.087–2.01)	0.70 (0.068–8.92)	1.21 (0–6.53)
IL23	0.08 (0.06–0.22)	0.00 (0–0.325)	0.98 (0–1.68)	0.00	0.03 (0–5.16)
IL25	0.02 (0–0.04)	0.01 (0.005–0.05)	0.07 (0.002–0.128)	0.00 (0–0.016)	0.00 (0–0.003)
IL31	0.85 (0–2.08)	0.78 (0.30–2.15)	1.72 (0.067–2.68)	0.00 (0–0.034)^†^	0.06 (0–5.12)
IL33	16.54 (11.10–26.40)	55.07 (8.85–95.2)	25.88 (11.5–57.8)	168.60 (60.0–232.0)	80.03 (9.86–649.0)
IFN-*γ*	0.06 (0.0225–0.132)	0.04 (0.019–0.095)	0.23 (0.051–1.25)^*∗*^	0.01 (0–0.019)	0.00 (0–0.66)
sCD40L	0.16 (0.045–0.363)	0.30 (0.170–2.01)	0.63 (0.25–0.924)	0.25 (0.024–1.44)	0.08 (0–2.08)
TNF-*α*	0.01 (0–0.04)	0.23 (0.182–0.48)^†^	0.20 (0.051–0.575)	10.88 (0.795–23.0)^†^	53.73 (0.55–68.9)

Data are presented as median (25–75). ^†^Significant difference in comparison with healthy control (*p* < 0.05). ^*∗*^Significant difference in comparison with the level before the treatment, *p* < 0.05. *Note.* L28 patient has not given consent to provide a sample of tumor tissue treatment.

## Data Availability

The authors confirm that the data supporting the findings of this study are available within the article and its supplementary materials.

## Supplementary Materials

Supplementary Materials Table 1S. Cytokine concentrations in the tumor tissue and surrounding tissue of patients and healthy controls before treatment. Table 2S. PUVA and NB-UVB efficacy in patients with mycosis fungoides. Table 3S. Cytokine concentrations in the tumor tissue of patients after PUVA and NB-UVB therapy.
