# Supplementary material for: Effect of PUVA and NB-UVB Therapy on the Skin Cytokine Profile in Patients with Mycosis Fungoides
Source: J Oncol. 2022 Feb 21;2022:3149293. doi: 10.1155/2022/3149293 (PMC8885178; doi:10.1155/2022/3149293)
Supplement: Supplementary Materials — Table 1S. Cytokine concentrations in the tumor tissue and surrounding tissue of patients and healthy controls before treatment. Table 2S. PUVA and NB-UVB efficacy in patients with mycosis fungoides. Table 3S. Cytokine concentrations in the tumor tissue of patients after PUVA and NB-UVB therapy. [file 3149293.f1.zip › 3149293.f1/Table 1S (1).docx]

Table 1S. Cytokine concentrations in the tumor tissue and surrounding tissue of patients and healthy controls before treatment

| Patient | Group | **IL1b** | **IL4** | **IL6** | **IL10** | **IL17A** | **IL17F** | **IL21** | **IL22** | **IL23** | **IL25** | **IL31** | **IL33** | **IFNg** | **sCD40L** | **TNFa** |  |
| --- | --- | --- | --- | --- | --- | --- | --- | --- | --- | --- | --- | --- | --- | --- | --- | --- | --- |
| L1 | Surrounding tissue | 0,451 | 1,748 | 0,521 | 0,168 | 0,349 | 0,851 | 1,394 | 0,584 | 0,932 | 0,084 | 1,610 | 17,380 | 1,697 | 0,605 | 0,103 |  |
| L4 | Surrounding tissue | 0,484 | 0,570 | 0,004 | 0,000 | 0,000 | 0,000 | 0,000 | 0,017 | 0,000 | 0,002 | 0,060 | 4,092 | 0,048 | 0,134 | 0,000 |  |
| L5 | Surrounding tissue | 0,773 | 1,298 | 0,023 | 0,000 | 0,217 | 0,000 | 0,453 | 0,293 | 0,000 | 0,042 | 0,120 | 6,976 | 0,118 | 0,299 | 0,017 |  |
| L6 | Surrounding tissue | 0,011 | 2,831 | 0,027 | 0,000 | 0,132 | 0,363 | 1,738 | 0,001 | 0,323 | 0,015 | 0,093 | 8,850 | 0,022 | 0,273 | 0,012 |  |
| L13 | Surrounding tissue | 0,75 | 0,62 | 0,05 | 0,00 | 0,00 | 0,00 | 0,00 | 0,00 | 0,00 | 0,00 | 0,00 | 182,21 | 0,00 | 20,71 | 99,00 |  |
| L14 | Surrounding tissue | 0,74 | 0,04 | 0,00 | 0,00 | 0,01 | 0,00 | 0,14 | 0,00 | 0,00 | 0,00 | 0,00 | 70,49 | 0,00 | 0,19 | 0,01 |  |
| L18 | Surrounding tissue | 4,74 | 0,39 | 0,03 | 0,41 | 0,25 | 0,00 | 1,61 | 0,72 | 0,00 | 0,06 | 1,41 | 561,60 | 0,15 | 0,50 | 0,31 |  |
| L22 | Surrounding tissue | 0,00 | 0,12 | 0,00 | 0,00 | 0,05 | 0,00 | 0,13 | 0,05 | 0,00 | 0,00 | 0,00 | 329,12 | 0,00 | 0,21 | 0,29 |  |
| L23 | Surrounding tissue | 6,03 | 0,01 | 0,21 | 0,12 | 0,60 | 1,05 | 2,04 | 2,72 | 0,00 | 0,15 | 7,56 | 3,85 | 0,73 | 0,03 | 0,26 |  |
| L24 | Surrounding tissue | 0,97 | 0,00 | 0,00 | 0,00 | 0,02 | 0,00 | 0,17 | 0,07 | 0,00 | 0,00 | 0,00 | 36,20 | 0,02 | 0,00 | 0,01 |  |
| L26 | Surrounding tissue | 0,42 | 0,16 | 0,03 | 0,20 | 0,11 | 0,66 | 10,10 | 0,92 | 2,02 | 0,10 | 3,38 | 1,64 | 0,76 | 0,80 | 0,27 |  |
| L27 | Surrounding tissue | 0,04 | 0,15 | 0,04 | 0,06 | 0,13 | 0,21 | 5,32 | 0,60 | 0,82 | 0,07 | 1,20 | 17,52 | 0,26 | 0,45 | 0,28 |  |
| L28 | Surrounding tissue | 0,76 | 0,28 | 0,03 | 0,13 | 0,20 | 0,58 | 6,44 | 0,83 | 1,30 | 0,11 | 2,72 | 30,91 | 0,37 | 1,06 | 0,42 |  |
| L29 | Surrounding tissue | 0,03 | 0,11 | 0,01 | 0,05 | 0,09 | 0,00 | 0,47 | 1,84 | 0,00 | 0,04 | 0,74 | 15,34 | 0,09 | 0,48 | 0,18 |  |
| L2 | Surrounding tissue | 0,096 | 0,412 | 0,000 | 0,000 | 0,000 | 0,000 | 0,000 | 0,000 | 0,000 | 0,002 | 0,022 | 0,591 | 0,000 | 0,085 | 0,000 |  |
| L8 | Surrounding tissue | 0,58 | 0,68 | 0,00 | 0,00 | 0,00 | 0,00 | 0,00 | 0,00 | 0,00 | 0,00 | 0,00 | 140,20 | 0,00 | 0,00 | 19,38 |  |
| L9 | Surrounding tissue | 0,22 | 0,00 | 0,00 | 0,00 | 0,00 | 0,00 | 0,00 | 0,03 | 0,00 | 0,00 | 0,00 | 11,94 | 0,00 | 0,00 | 0,00 |  |
| L10 | Surrounding tissue | 0,38 | 3,44 | 0,00 | 0,00 | 0,00 | 0,00 | 0,00 | 0,00 | 0,00 | 0,00 | 0,00 | 871,79 | 0,00 | 57,69 | 100,22 |  |
| L21 | Surrounding tissue | 2,07 | 0,11 | 0,03 | 0,19 | 0,14 | 0,50 | 0,86 | 0,69 | 7,12 | 0,05 | 3,78 | 91,45 | 0,14 | 0,25 | 0,16 |  |
| L1 | Tumor tissue | 0,388 | 2,314 | 0,538 | 0,172 | 0,444 | 0,689 | 1,100 | 1,513 | 0,605 | 0,051 | 1,700 | 39,803 | 1,121 | 0,836 | 0,164 |  |
| L3 | Tumor tissue | 0,163 | 2,368 | 4,696 | 0,091 | 1,426 | 0,530 | 1,350 | 0,109 | 0,000 | 0,155 | 0,430 | 46,615 | 0,249 | 2,554 | 0,229 |  |
| L4 | Tumor tissue | 0,103 | 1,626 | 0,590 | 0,094 | 0,100 | 0,231 | 0,900 | 5,347 | 0,045 | 0,012 | 0,179 | 10,140 | 0,052 | 2,548 | 0,157 |  |
| L5 | Tumor tissue | 0,012 | 0,940 | 0,016 | 0,000 | 0,124 | 0,000 | 0,157 | 1,433 | 0,000 | 0,001 | 0,048 | 7,174 | 0,000 | 0,171 | 0,016 |  |
| L6 | Tumor tissue | 0,015 | 0,457 | 0,020 | 0,000 | 0,029 | 0,051 | 0,161 | 0,012 | 0,000 | 0,010 | 0,041 | 4,319 | 0,019 | 0,195 | 0,018 |  |
| L7 | Tumor tissue | 1,09 | 0,88 | 0,22 | 0,00 | 0,00 | 0,00 | 0,00 | 0,03 | 0,00 | 0,00 | 5,25 | 202,61 | 0,13 | 8,37 | 27,46 |  |
| L13 | Tumor tissue | 0,79 | 1,72 | 1,79 | 0,00 | 0,83 | 6,28 | 0,00 | 0,00 | 0,00 | 0,00 | 9,32 | 79,35 | 0,00 | 26,37 | 64,81 |  |
| L14 | Tumor tissue | 0,73 | 0,07 | 0,00 | 0,00 | 0,02 | 0,11 | 0,19 | 2,66 | 0,00 | 0,01 | 1,27 | 109,30 | 0,03 | 0,30 | 0,28 |  |
| L18 | Tumor tissue | 1,05 | 0,16 | 0,02 | 0,16 | 0,15 | 0,00 | 0,35 | 0,38 | 3,83 | 0,02 | 1,01 | 172,21 | 0,06 | 0,17 | 0,23 |  |
| L22 | Tumor tissue | 0,98 | 0,00 | 0,00 | 0,00 | 0,01 | 0,00 | 0,12 | 0,28 | 0,00 | 0,00 | 0,00 | 81,11 | 0,00 | 0,20 | 0,54 |  |
| L23 | Tumor tissue | 4,74 | 0,14 | 0,00 | 0,00 | 0,09 | 0,00 | 0,65 | 0,65 | 0,00 | 0,05 | 7,91 | 60,32 | 0,04 | 0,51 | 0,20 |  |
| L24 | Tumor tissue | 0,94 | 0,12 | 0,01 | 0,00 | 0,02 | 0,00 | 0,12 | 0,04 | 0,00 | 0,01 | 0,78 | 111,90 | 0,02 | 0,13 | 0,42 |  |
| L26 | Tumor tissue | 0,05 | 0,03 | 0,03 | 0,03 | 0,07 | 0,18 | 3,67 | 1,80 | 0,94 | 0,05 | 0,70 | 3,65 | 0,04 | 0,00 | 0,21 |  |
| L27 | Tumor tissue | 0,10 | 0,49 | 0,06 | 0,10 | 0,20 | 0,00 | 13,93 | 1,13 | 1,81 | 0,16 | 2,60 | 55,07 | 0,47 | 1,48 | 1,01 |  |
| L28 | Tumor tissue | 0,53 | 0,49 | 0,06 | 0,26 | 0,20 | 1,01 | 11,75 | 1,15 | 2,82 | 0,13 | 3,64 | 35,58 | 0,67 | 1,79 | 2,55 |  |
| L29 | Tumor tissue | 0,02 | 0,04 | 0,01 | 0,00 | 0,04 | 0,00 | 1,02 | 2,35 | 0,00 | 0,02 | 0,42 | 7,55 | 0,05 | 0,12 | 0,21 |  |
| L2 | Tumor tissue | 0,024 | 0,636 | 0,006 | 0,000 | 0,007 | 0,000 | 0,190 | 0,010 | 0,000 | 0,005 | 0,045 | 2,442 | 0,015 | 0,095 | 0,006 |  |
| L8 | Tumor tissue | 0,18 | 1,40 | 0,58 | 0,00 | 0,00 | 1,02 | 0,00 | 26,29 | 0,00 | 0,00 | 0,00 | 338,63 | 0,00 | 0,00 | 37,14 |  |
| L9 | Tumor tissue | 0,10 | 0,73 | 0,00 | 0,00 | 0,00 | 0,00 | 0,00 | 11,51 | 0,00 | 0,00 | 0,00 | 133,44 | 0,00 | 0,00 | 19,21 |  |
| L10 | Tumor tissue | 0,10 | 0,83 | 0,00 | 0,00 | 0,00 | 0,00 | 0,00 | 0,00 | 0,00 | 0,00 | 0,00 | 203,76 | 0,00 | 13,58 | 24,25 |  |
| L21 | Tumor tissue | 1,32 | 0,14 | 0,00 | 0,00 | 0,06 | 0,00 | 0,26 | 0,24 | 0,00 | 0,02 | 0,00 | 241,93 | 0,02 | 0,40 | 0,21 |  |
| Z1 | Control | 0,11 | 0,07 | 0 | 0,02 | 0,12 | 0,18 | 0 | 0,4 | 0,08 | 0,04 | 1,7 | 18,72 | 0,1 | 0,26 | 0,03 |  |
| Z2 | Control | 0,06 | 0,15 | 0,02 | 0,1 | 0,21 | 0,42 | 0,46 | 0,52 | 0,64 | 0,04 | 3,23 | 49,42 | 0,23 | 0,67 | 0,07 |  |
| Z3 | Control | 0 | 0 | 0 | 0 | 0 | 0,17 | 0 | 0 | 0,08 | 0 | 0 | 1,28 | 0 | 0 | 0 |  |
| Z4 | Control | 0,03 | 0 | 0 | 0 | 0,02 | 0 | 0 | 0,22 | 0 | 0 | 0 | 14,35 | 0,03 | 0,06 | 0 |  |
| Note: Patients L3 and L7 have not given consent to provide a sample of unaffected skin | | | | | | | | | | | | | | | | | |
