# Supplementary material for: Effect of PUVA and NB-UVB Therapy on the Skin Cytokine Profile in Patients with Mycosis Fungoides
Source: J Oncol. 2022 Feb 21;2022:3149293. doi: 10.1155/2022/3149293 (PMC8885178; doi:10.1155/2022/3149293)
Supplement: Supplementary Materials — Table 1S. Cytokine concentrations in the tumor tissue and surrounding tissue of patients and healthy controls before treatment. Table 2S. PUVA and NB-UVB efficacy in patients with mycosis fungoides. Table 3S. Cytokine concentrations in the tumor tissue of patients after PUVA and NB-UVB therapy. [file 3149293.f1.zip › 3149293.f1/Table 2S.docx]

Table 2S. PUVA and NB-UVB efficacy in patients with mycosis fungoides

| **Therapy** | **№** | **Patient** | **Age** | **Diagnosis** | **Stage** | **Duration of the disease (years)** | **Number of procedures performed** | **Total dose, J / cm2** | **mSWAT** | **BSA** | **BSA** | **% of BSA decrease** | **mSWAT** | **% of mSWAT decrease** | **Effect** |
| --- | --- | --- | --- | --- | --- | --- | --- | --- | --- | --- | --- | --- | --- | --- | --- |
| **NB-UVB** | 1 | L2 | 48 | Mycosis fungoides | IB | 4,5 | 40 | 20,05 | 11 | 8 | 0 | 100 | 0 | 100 | CR |
|  | 2 | L8 | 71 | Mycosis fungoides | IB | 30 | 25 | 18,17 | 34,3 | 28,8 | 10,3 | 69,9 | 10,3 | 69,9 | PR |
|  | 3 | L9 | 57 | Mycosis fungoides | IIA | 17 | 13 | 2,1 | 101,8 | 56,4 | 41,6 | 26,2 | 59,7 | 41,3 | S |
|  | 4 | L10 | 28 | Mycosis fungoides | IB | 13 | 32 | 22,8 | 16 | 11 | 8,5 | 22,7 | 10,5 | 34,3 | S |
|  | 5 | L12 | 67 | Mycosis fungoides | IB | 3 | 20 | 19,8 | 105 | 63 | 44,2 | 70 | 55,2 | 47,4 | PR |
|  | 6 | L21 | 62 | Mycosis fungoides | IIA | 8 | 38 | 28,95 | 44,1 | 35 | 7 | 80 | 7 | 84,1 | PR |
|  | 7 | L28 | 32 | Mycosis fungoides | IB | 14 | 30 | 3,2 | 14,5 | 12,5 | 10 | 16,6 | 12,5 | 13,7 | Abs+undesirable events |
|  | 8 | L30 | 68 | Mycosis fungoides | IB | 7 | 33 | 22,8 | 13,5 | 9 | 2,3 | 74,4 | 2,3 | 82,9 | PR |
|  | 9 | L36 | 72 | Mycosis fungoides | IB | 8 | 35 | 23,02 | 17,5 | 12,5 | 4,5 | 64 | 6 | 65,7 | PR |
|  | 10 | L37 | 53 | Mycosis fungoides | IIA | 22 | 36 | 17,09 | 26 | 21 | 4,9 | 76,7 | 5,6 | 78,5 | PR |
| **PUVA** | 11 | L1 | 63 | Mycosis fungoides | IB | 9 | 20 | 61 | 12 | 9 | 2,8 | 68,8 | 3,1 | 74,1 | PR |
|  | 12 | L3 | 38 | Mycosis fungoides | IIA | 10 | 17 | 31,5 | 23,5 | 15 | 12 | 20 | 17 | 72,3 | S |
|  | 13 | L4 | 68 | Mycosis fungoides | IIB | 7 | 16 | 32,5 | 64,8 | 37,3 | 27,6 | 26 | 46,3 | 28,3 | S |
|  | 14 | L5 | 62 | Mycosis fungoides | IIA | 21 | 30 | 124,5 | 29,5 | 16,4 | 7,8 | 52,4 | 8,4 | 71,5 | PR |
|  | 15 | L6 | 65 | Mycosis fungoides | IIA | 6 | 40 | 79,69 | 38,1 | 24,3 | 4,4 | 81,8 | 7,4 | 80,6 | PR |
|  | 16 | L7 | 38 | Mycosis fungoides | IB | 5 | 31 | 129,2 | 17,8 | 14,8 | 0,7 | 95,2 | 0,7 | 96 | PR |
|  | 17 | L13 | 63 | Mycosis fungoides | IB | 14 | 19 | 35 | 74,5 | 49 | 21 | 57,1 | 35 | 53 | PR |
|  | 18 | L14 | 65 | Mycosis fungoides | IB | 35 | 20 | 36,75 | 25 | 12 | 5,3 | 55,8 | 5,3 | 78,8 | PR |
|  | 19 | L18 | 32 | Mycosis fungoides | IB | 3 | 18 | 32,35 | 14,3 | 7,9 | 1 | 87,3 | 1 | 93 | PR |
|  | 20 | L22 | 58 | Mycosis fungoides | IIA | 10 | 38 | 171,5 | 58,6 | 35,5 | 0,3 | 99,1 | 0,6 | 98,9 | CR |
|  | 21 | L23 | 66 | Mycosis fungoides | IB | 30 | 40 | 187,75 | 13,1 | 7,8 | 3 | 61,5 | 4 | 69,4 | PR |
|  | 22 | L24 | 54 | Mycosis fungoides (CD30+) | IIA | 30 | 40 | 159,5 | 12 | 8 | 2,5 | 68,7 | 2,5 | 79 | PR |
|  | 23 | L26 | 74 | Mycosis fungoides | IIA | 22 | 36 | 218,25 | 99,4 | 64 | 12,5 | 80,4 | 20,5 | 79,3 | PR |
|  | 24 | L27 | 67 | Mycosis fungoides | IIA | 3 | 40 | 156,25 | 26 | 22 | 7,5 | 65,9 | 7,5 | 71,1 | PR |
|  | 25 | L29 | 54 | Mycosis fungoides | IIA | 9 | 37 | 161,5 | 68,5 | 55 | 15,5 | 71,8 | 20,5 | 70 | PR |
|  | 26 | L32 | 69 | Mycosis fungoides | IIA | 2 | 12 | 23 | 39 | 24 | 24 | 0 | 32 | 17,9 | Progression |
|  | 27 | L33 | 58 | Mycosis fungoides | IB | 10 | 36 | 103,6 | 16 | 10 | 4,8 | 52 | 6,6 | 58,7 | PR |
| CR – complete remission, PR – partial remission, S – stabilization, Abs – absence of effect | | | | | | | | | | | | | | | |
