# Supplementary material for: Effect of PUVA and NB-UVB Therapy on the Skin Cytokine Profile in Patients with Mycosis Fungoides
Source: J Oncol. 2022 Feb 21;2022:3149293. doi: 10.1155/2022/3149293 (PMC8885178; doi:10.1155/2022/3149293)
Supplement: Supplementary Materials — Table 1S. Cytokine concentrations in the tumor tissue and surrounding tissue of patients and healthy controls before treatment. Table 2S. PUVA and NB-UVB efficacy in patients with mycosis fungoides. Table 3S. Cytokine concentrations in the tumor tissue of patients after PUVA and NB-UVB therapy. [file 3149293.f1.zip › 3149293.f1/Table 3S (1).docx]

Table 3S. Cytokine concentrations in the tumor tissue of patients after PUVA and NB-UVB therapy

| Patient | Treatment | **IL1b** | **IL4** | **IL6** | **IL10** | **IL17A** | **IL17F** | **IL21** | **IL22** | **IL23** | **IL25** | **IL31** | **IL33** | **IFNg** | **sCD40L** | **TNFa** |
| --- | --- | --- | --- | --- | --- | --- | --- | --- | --- | --- | --- | --- | --- | --- | --- | --- |
| L1 | PUVA | 0.189 | 2.795 | 0.962 | 0.365 | 0.522 | 1.477 | 2.308 | 1.173 | 1.945 | 0.145 | 3.399 | 17.690 | 3.174 | 0.887 | 0.164 |
| L3 | PUVA | 0.220 | 1.069 | 0.752 | 0.000 | 0.020 | 0.000 | 0.000 | 0.000 | 0.000 | 0.005 | 0.080 | 25.876 | 0.066 | 1.114 | 0.044 |
| L4 | PUVA | 0.114 | 0.253 | 0.039 | 0.000 | 0.029 | 0.000 | 0.122 | 0.336 | 0.000 | 0.000 | 0.200 | 2.697 | 0.000 | 0.130 | 0.026 |
| L5 | PUVA | 0.038 | 0.917 | 0.012 | 0.000 | 0.033 | 0.000 | 0.229 | 0.012 | 0.000 | 0.004 | 0.054 | 7.595 | 0.035 | 0.114 | 0.006 |
| L6 | PUVA | 0.037 | 3.187 | 0.200 | 0.104 | 0.271 | 0.991 | 3.175 | 0.162 | 1.125 | 0.083 | 0.258 | 62.182 | 0.235 | 0.529 | 0.057 |
| L7 | PUVA | 0.41 | 0.92 | 0.00 | 0.00 | 0.00 | 0.00 | 0.00 | 0.00 | 0.00 | 0.00 | 0.00 | 234.67 | 0.00 | 9.23 | 25.75 |
| L13 | PUVA | 0.40 | 0.00 | 0.00 | 0.81 | 0.00 | 0.26 | 0.00 | 4.32 | 0.00 | 0.00 | 0.00 | 65.83 | 5.23 | 0.00 | 39.79 |
| L14 | PUVA | 1.59 | 0.00 | 0.11 | 0.00 | 0.05 | 0.00 | 0.19 | 0.00 | 0.00 | 0.00 | 0.00 | 32.87 | 0.03 | 0.00 | 0.00 |
| L18 | PUVA | 5.95 | 0.17 | 0.03 | 0.23 | 0.49 | 0.00 | 2.04 | 2.66 | 0.00 | 0.11 | 2.12 | 73.96 | 0.48 | 0.37 | 0.17 |
| L22 | PUVA | 0.02 | 0.36 | 0.02 | 0.10 | 0.08 | 0.40 | 6.54 | 0.57 | 0.98 | 0.07 | 1.72 | 31.95 | 0.15 | 0.68 | 0.26 |
| L23 | PUVA | 5.70 | 0.71 | 0.11 | 0.40 | 0.41 | 1.89 | 27.89 | 2.70 | 5.99 | 0.40 | 11.05 | 22.07 | 1.62 | 2.93 | 0.88 |
| L24 | PUVA | 0.09 | 0.79 | 0.08 | 0.41 | 0.21 | 1.03 | 14.16 | 1.36 | 3.14 | 0.17 | 3.13 | 53.42 | 0.88 | 0.75 | 0.59 |
| L26 | PUVA | 0.27 | 0.18 | 0.05 | 0.10 | 0.14 | 0.41 | 6.25 | 0.87 | 1.39 | 0.10 | 2.22 | 13.42 | 0.20 | 0.55 | 0.23 |
| L27 | PUVA | 0.10 | 0.16 | 0.04 | 0.15 | 0.11 | 0.46 | 5.95 | 0.46 | 1.41 | 0.07 | 2.04 | 9.51 | 0.25 | 0.63 | 0.20 |
| L29 | PUVA | 0.05 | 0.25 | 0.08 | 0.73 | 0.38 | 0.67 | 25.74 | 2.79 | 4.01 | 0.21 | 3.69 | 3.14 | 3.33 | 0.96 | 0.56 |
| L2 | NB-UVB | 0.053 | 1.268 | 0.006 | 0.000 | 0.024 | 0.000 | 0.202 | 0.000 | 0.026 | 0.003 | 0.060 | 7.348 | 0.000 | 0.076 | 0.002 |
| L8 | NB-UVB | 2.76 | 0.00 | 0.00 | 0.00 | 0.00 | 0.00 | 0.00 | 0.00 | 0.00 | 0.00 | 0.00 | 80.03 | 0.00 | 0.00 | 53.73 |
| L9 | NB-UVB | 3.82 | 5.71 | 0.00 | 0.00 | 0.00 | 0.00 | 0.00 | 10.92 | 0.00 | 0.00 | 0.00 | 1312.52 | 0.00 | 0.00 | 109.07 |
| L10 | NB-UVB | 0.20 | 4.30 | 0.11 | 0.88 | 0.36 | 4.13 | 17.06 | 6.53 | 11.03 | 0.00 | 102.75 | 649.12 | 8.10 | 27.07 | 68.94 |
| L21 | NB-UVB | 0.29 | 0.49 | 0.08 | 0.43 | 0.24 | 1.37 | 17.37 | 1.21 | 5.16 | 0.22 | 5.12 | 9.86 | 0.66 | 2.08 | 0.55 |
| L28 | NB-UVB |  |  |  |  |  |  |  |  |  |  |  |  |  |  |  |
| Cytokine level is expressed as pg/mg of tissue  Note: L28 patient has not given consent to provide a sample of tumor tissue after treatment | | | | | | | | | | | | | | | | |
